# Quantitative evaluation of cell death response *in vitro* and *in vivo* using conventional-frequency ultrasound

**DOI:** 10.18632/oncoscience.235

**Published:** 2015-09-03

**Authors:** Ali Sadeghi-Naini, Stephanie Zhou, Mehrdad J. Gangeh, Zahra Jahedmotlagh, Omar Falou, Shawn Ranieri, Muhammad Azrif, Anoja Giles, Gregory J. Czarnota

**Affiliations:** ^1^ Physical Sciences, Sunnybrook Research Institute, Sunnybrook Health Sciences Centre, Toronto, ON, Canada; ^2^ Department of Radiation Oncology, Odette Cancer Centre, Sunnybrook Health Sciences Centre, Toronto, ON, Canada; ^3^ Department of Medical Biophysics, Faculty of Medicine, University of Toronto, Toronto, ON, Canada; ^4^ Department of Radiation Oncology, Faculty of Medicine, University of Toronto, Toronto, ON, Canada

**Keywords:** apoptosis, quantitative ultrasound, cancer therapy, treatment response monitoring, personalized medicine

## Abstract

Previous studies using high-frequency ultrasound have suggested that radiofrequency (RF) spectral analysis can be used to quantify changes in cell morphology to detect cell death response to therapy non-invasively. The study here investigated this at conventional-frequencies, frequently used in clinical settings.

Spectral analysis was performed using ultrasound RF data collected with a clinical ultrasound platform. Acute myeloid leukemia (AML-5) cells were exposed to cisplatinum for 0–72 hours *in vitro* and prepared for ultrasound data collection. Preclinical *in vivo* experiments were also performed on AML-5 tumour-bearing mice receiving chemotherapy.

The mid-band fit (MBF) spectral parameter demonstrated an increase of 4.4 ± 1.5 dBr for *in vitro* samples assessed 48 hours after treatment, a statistically significant change (*p* < 0.05) compared to control. Further, *in vitro* concentration-based analysis of a mixture of apoptotic and untreated cells indicated a mean change of 10.9 ± 2.4 dBr in MBF between 0% and 40% apoptotic cell mixtures. Similar effects were reproduced *in vivo* with an increase of 4.6 ± 0.3 dBr in MBF compared to control, for tumours with considerable apoptotic areas within histological samples. The alterations in the size of cells and nuclei corresponded well with changes measured in the quantitative ultrasound (QUS) parameters.

## INTRODUCTION

Assessing the efficacy of cancer treatments on an individual patient basis is presently limited from a clinical perspective. This is mainly because of the fact that currently even imaging-based monitoring of cancer therapy effects is frequently performed at macroscopic levels, relying on the standard clinical practice to measure the size of tumour [1]. Changes in tumour size, however, can take several weeks to months to become apparent, and do not always occur even when the treatment is effective [2, 3]. In order to overcome this problem, cancer therapy response monitoring at microscopic physiological levels has gained much attention in cancer research and imaging [3–5]. Functional imaging techniques are thus commonly proposed based on the observations that development of response to cancer therapy introduces micro-structural, morphological, and functional alterations within tumour, which can be detected non-invasively early on after the start of treatment using different imaging modalities [2].

Different imaging modalities including positron emission tomography (PET) [6], single photon emission computed tomography (SPECT) [7], and magnetic resonance imaging (MRI) [8] have been investigated for the evaluation of responses to cancer treatment, based on changes in cell metabolism and blood perfusion [5], or the development of apoptotic cell death within tumours [9]. However, these methods have two drawbacks, *i.e*., being presently expensive and requiring exogenous contrast agents frequently. The agents' costs, radiation dose involved, and the potential for side effects including allergic reactions limits their applicability as a standard method to be used routinely in clinic. Amongst other imaging techniques, quantitative ultrasound (QUS) techniques coupled with high-frequency high-resolution ultrasound have shown promise in detecting development of cell death in response to cancer therapies [3, 4, 10, 11]. Such techniques, if translated into conventional clinically-relevant frequency ranges, could provide a cost-effective and rapid framework to assess cancer therapy effects. These methods also alleviate the requirement for injecting external contrast agents since in these methods, the alterations in bio-acoustic properties of dying cancerous cells are the main source of changes in imaging contrast [10, 12].

In this context, early investigations combining well controlled biological experimentation and high-frequency QUS techniques have provided valuable information to detect and potentially quantify cell death [10, 11]. Ultrasound has been used successfully as a clinical diagnostic tool for many years because it is safe, real-time, non-invasive, and relatively inexpensive. Ultrasonic scattering in biological tissues is a complex process. It is primarily affected by the acoustic impedance mismatches and the size and density of scattering structures within tissue, in relation to the ultrasound wavelength. The application of ultrasound in cancer imaging is based on the foundation that pathological or therapeutic processes alter physical characteristics of tissue such as compressibility, density, and geometry of potential scatterers, and these alterations cause observable changes in acoustic scattering properties. Such physical characteristics of ultrasound make it a very attractive tool to monitor changes in tumours in response to treatment. Based on these principles, high-frequency ultrasound has been used to monitor structural changes at the cellular level *in vitro* and *in vivo* (described further below).

High-frequency ultrasound has been used to detect apoptosis, necrosis and other forms of cell death *in vitro*, *in situ*, and *in vivo* [10, 11, 13–15]. It was demonstrated that cell death could be detected *in vivo* using high-frequency QUS spectral analysis techniques in preclinical animal models, where xenograft tumours were treated using a number of different modalities. The first preclinical use of high-frequency QUS spectral analysis for monitoring cancer therapy effects was to evaluate responses of xenografted melanoma tumours to photodynamic therapy (PDT) *in vivo* [15]. Banihashemi *et al.* observed a time-dependent increase in QUS spectral parameters, namely mid-band fit (MBF) and spectral slope, after treatment. The MBF is a measure of ultrasound backscatter power, and the spectral slope parameter has been demonstrated to be influenced by the size of effective acoustic scatterers [16, 17]. One other parameter, the 0-MHz intercept can be related mathematically to the concentration of acoustic scatterers. The observed increases in these spectral parameters correlated with morphologic findings, indicating increases in apoptotic cell death after PDT. Analyses of changes in spectral slope strongly correlated with changes in mean nuclear size over time, associated with apoptosis induced by the therapy. In another study, Vlad *et al*. used high-frequency ultrasound in a similar manner to track the responses of xenograft tumours *in vivo* to radiotherapy [14]. Three mouse models grafted with head and neck carcinomas (FaDu, C666–1, and Hep-2) exhibited large hyperechoic regions after radiotherapy. The ultrasound integrated backscatter increased by 6.5 to 8.2 dBr (*p* < 0.001) and spectral slopes increased from 0.77 to 0.90 dBr/MHz for C666–1 tumours and from 0.54 to 0.78 dBr/MHz for FaDu tumours (*p* < 0.05), compared with pre-irradiated tumours. The hyperechoic regions in the ultrasound images corresponded in histology to areas of cell death.

Whereas high-frequency ultrasound (20–50 MHz) has the main advantage of providing higher resolution compared with conventional-frequency ultrasound (1–20 MHz), it is limited by a low penetration depth, which makes it unsuitable for deeper structures such as those in the abdomen and in breast imaging. This limits the application of high-frequency ultrasound to research and very superficial tissues such as the eye [18]. Conventional-frequency ultrasound penetrates deeper into body structures and hence is used with most clinical ultrasound equipment. Therefore, for a broad adoption of QUS methods in clinical applications for cancer response monitoring, the extension of these methods to conventional-frequency range is highly desired.

In this study, conventional-frequency (~7 MHz) QUS spectral analysis techniques were investigated for the assessment of cancer therapy effects *in vitro*, and *in vivo*, in order to demonstrate an efficacy for the non-invasive detection of cell death. Ultrasound data was collected at conventional frequencies *in vitro* from acute myeloid leukemia (AML-5) cell samples before and at different times after cisplatinum chemotherapy exposure. Experiments *in vivo* were conducted on AML-5 tumour-bearing mice undergoing chemotherapy and tumours were imaged before and after treatment. Ultrasound data were analyzed using spectral analysis techniques with normalized power spectra derived from ultrasound RF data. Time-based *in vitro* experiments indicated statistically significant changes in spectral parameters 48 hours after the administration of chemotherapy (*p* < 0.05). Moreover, concentration-based experiments here demonstrated an average increase of 10.9 ± 2.4 dBr in MBF between 0% and 40% mixtures of apoptotic and viable cells. Similar confirmatory results were obtained in *in vivo* experiments. Obtained results demonstrated that QUS techniques at conventional-frequencies can be applied to detect cell death in response to anti-cancer therapies. This work here therefore suggests good promise for the use of QUS techniques in a clinical setting for the monitoring treatment response in cancer patients [2].

## RESULTS

### *In vitro* experiments

**Figure 1:**
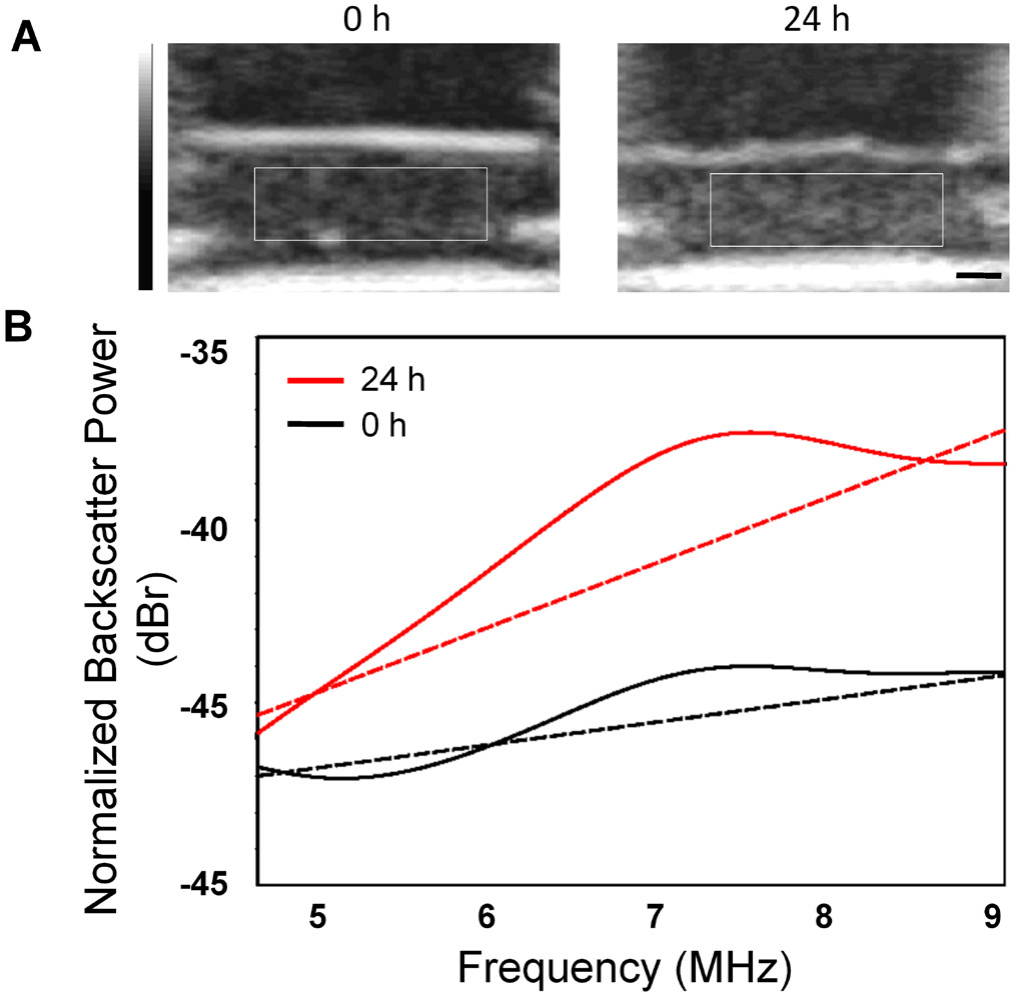
Representative ultrasound data acquired *in vitro* **A.** Conventional-frequency ultrasound B-mode images obtained from control and treated AML-5 cell samples. Rectangles demonstrate the ROIs for the spectral analysis. The scale bar represents ~ 1 mm. **B.** Normalized power spectra illustrating the typical disparity in intensity and shape of ultrasound backscatter power spectra collected from control and treated cell samples.

Exposure to the chemotherapeutic agent, cisplatinum, produced constant and reproducible changes in ultrasound backscatter and the derived spectral parameters as a function of time after exposure (described further below). To better illustrate these effects, Figure 1 shows representative ultrasound B-mode images and normalized power spectra from a control untreated and a treated cell sample, demonstrating an increase in echogenicity within cell samples 24 hours after treatment. This also affected the corresponding normalized power spectra where an increase in backscatter power was apparent across the entire bandwidth of the transducer. These changes in ultrasound backscatter and the spectral parameters during treatment are known to be linked to structural and morphological changes that occur throughout apoptosis in AML-5 cells [15].

**Figure 2:**
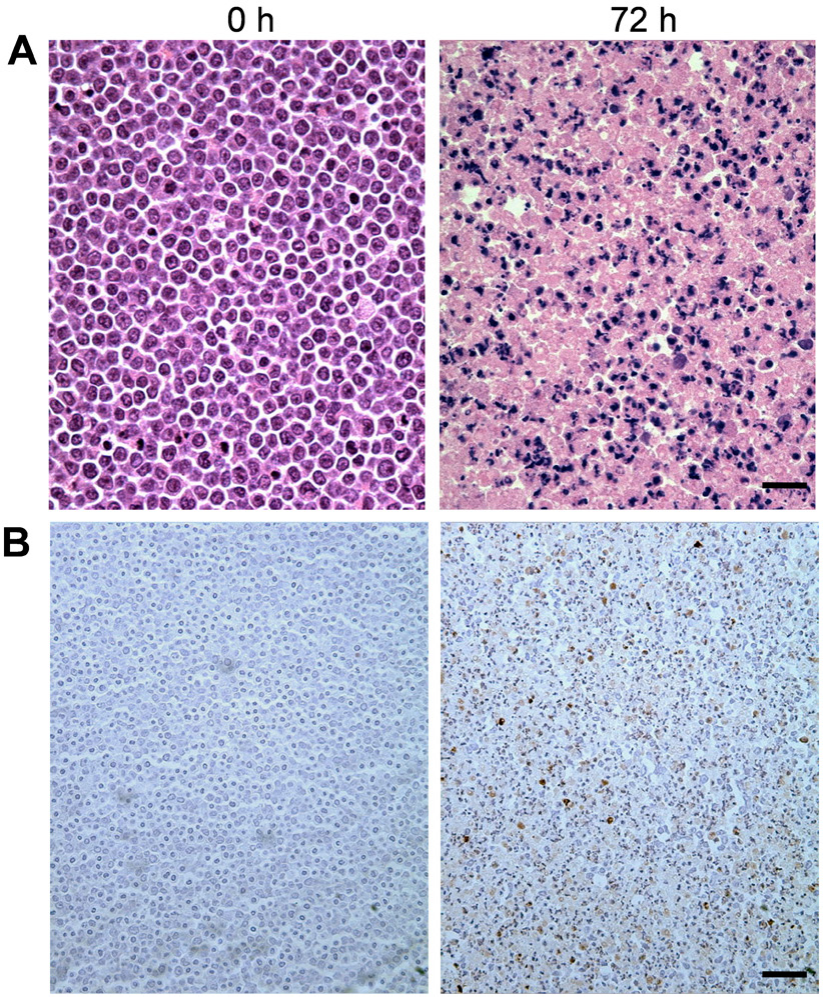
Representative light microscopy images of histology slides at 40× magnification from *in vitro* experiments **A.** H&E stained slides and **B.** TUNEL stained slides obtained from control and treated AML-5 cell samples. The scale bars in (A) and (B) represent ~ 20 μm and ~ 50 μm, respectively.

**Figure 3:**
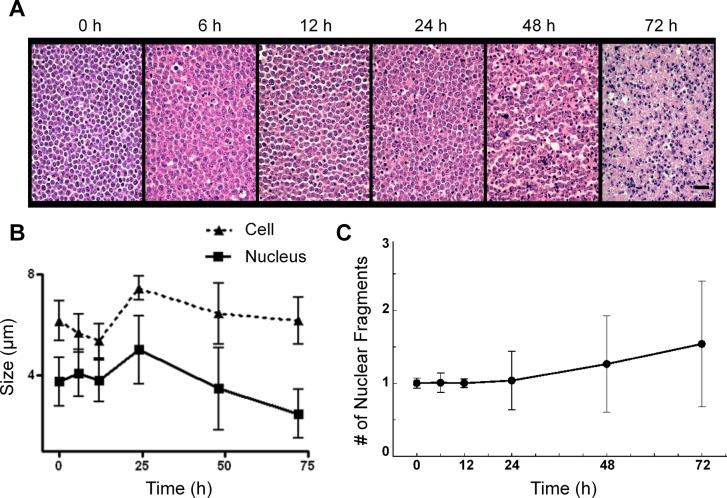
Results of time-dependent histological analysis on AML-5 cell samples *in vitro* **A.** Representative light microscopy images of H&E stained slides at 40× magnification, corresponding to 0, 6, 12, 24, 48 and 72 hours after the treatment. The scale bar represents ~ 20 μm. **B.** Average sizes of the cells and their nuclei measured before and at different times after chemotherapy exposure. **C.** Average number of nuclear fragments observed before and at different times after the treatment. Error bars represent ± one standard deviation.

Figure 2 demonstrates light microscopy images of H&E and TUNEL stained slides at high magnification, obtained from representative control and treated cell samples. Considerable changes were observed within the H&E slides in terms of cellular and nuclear structure and morphology after chemotherapy. A noteworthy point is the large number of cells without any nuclei or nuclear fragments at 72 hours after treatment, indicative of late-stage apoptosis. The TUNEL stained slides also confirmed that a considerable number of cells within the samples underwent apoptosis in response to chemotherapy. Figure 3 demonstrates results of time-dependent histological analysis on AML-5 cell samples. Figure 3A illustrates light microscopy images of the representative H&E stained slides of AML-5 cell samples for each specific experimental time after treatment. Structural and morphological alterations were evident in cells and nuclei throughout the apoptosis process in response to treatment. The number of cells with degraded nuclear material accounted for 46% of the total number of cells imaged at 72 hours after chemotherapy exposure. Figures 3B and 3C demonstrate the changes in the average size of the cells and nuclei, and the average number of nuclear fragments, respectively, as functions of time (0, 6, 12, 24, 48, and 72 hours) after exposure to cisplatinum. Initial increases were observed in the average size of cells and nuclei, before steady and more homogeneous size decreases after 24 hours of treatment. This implies substantial morphological changes in cells and nuclei such as cellular blebbing and nuclear coalescence at early stages after treatment, followed by cellular and nuclear condensation and fragmentation and structural disorganization at later stages, as hallmarks of apoptotic cell death. The mean nucleus:cell size ratio decreased from 0.7 at 0 hours to 0.4 at 72 hours. An increase was also noticed in the average number of nuclear fragments per cell over this time duration.

**Figure 4:**
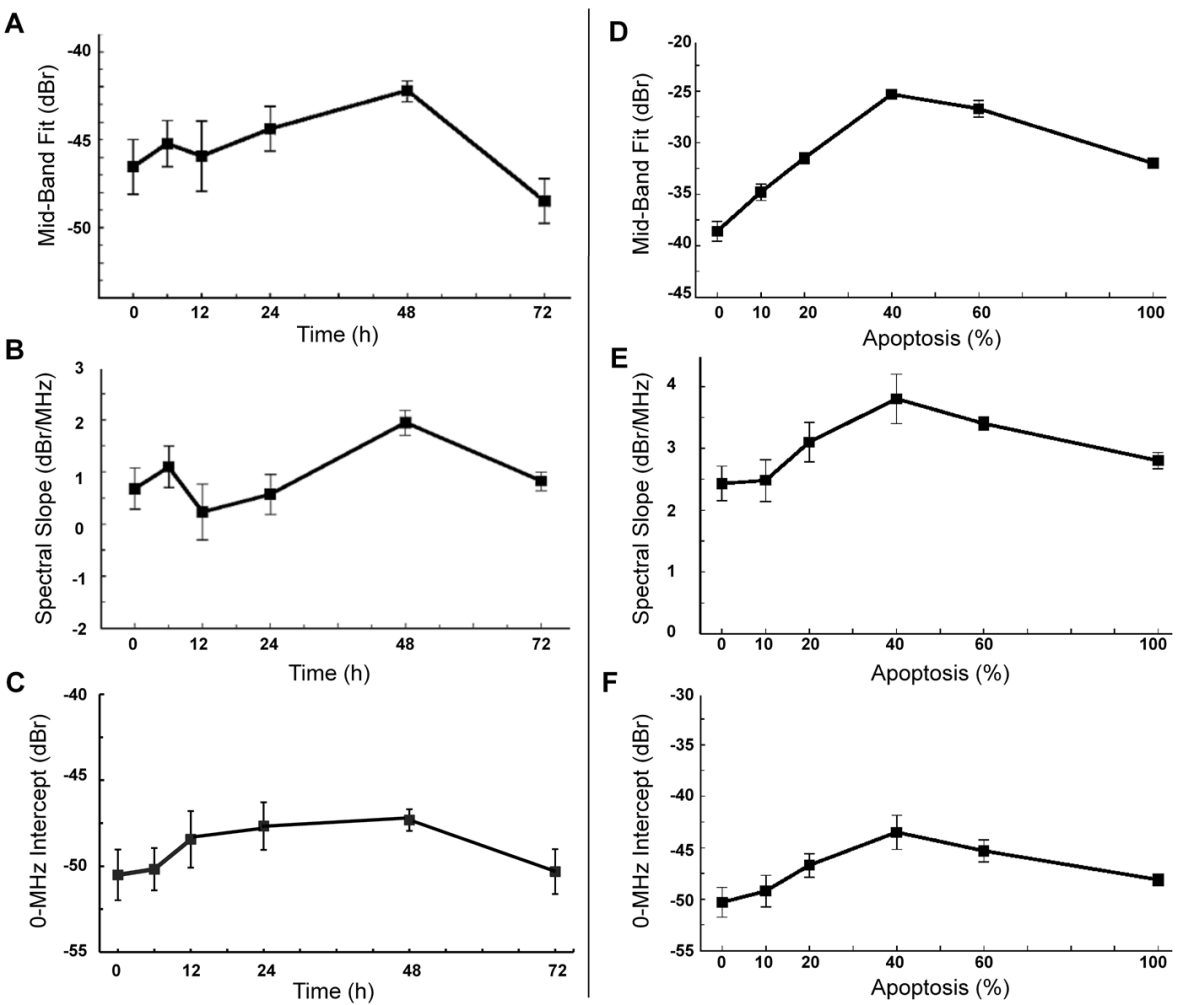
Average values of quantitative ultrasound spectral parameters obtained for AML-5 cell samples *in vitro* using ~7 MHz ultrasound Changes in the **A.** MBF, **B.** spectral slope and **C.** 0-MHz intercept parameters are shown at different times after chemotherapy exposure. Trends in the **D.** MBF, **E.** spectral slope and **F.** 0-MHz intercept parameters are demonstrated as functions of proportions of apoptotic cells mixed with untreated cells. Error bars represent ± one standard error.

Mean values obtained for QUS spectral parameters at different times after exposure to chemotherapy are presented in Figures 4A–4C. Figure 4A demonstrates increases in the MBF parameter (a measure of ultrasound backscatter power) up to 48 hours after treatment, very similar to the trend observed previously with high-frequency ultrasound [11]. Specifically, the average values obtained for MBF at 0, 24, 48, and 72 hours after treatment were −46.5 ± 1.4 dBr, −44.4 ± 1.3 dBr, −42.1 ± 0.6 dBr, and −48.5 ± 1.3 dBr, respectively. In particular, the MBF increased by 4.4 ± 1.5 dBr at 48 hours and decreased substantially at 72 hours after treatment. Statistical tests of significance (*t*-test, unpaired, two-sided) demonstrated the measured change at 48 hours to be significant (*p* < 0.05), compared to pre-treatment. Figure 4B presents the corresponding spectral slope parameter (influenced by effective scatterer size) which exhibited a similar trend. The spectral slope increased from 0.7 ± 0.5 dBr/MHz prior to treatment to 2.0 ± 0.2 dBr/MHz at 48 hours, and then decreased to 0.8 ± 0.1 dBr/MHz at 72 hours after treatment. Similarly, Figure 4C demonstrates mean increases in the 0-MHz intercept parameter (influenced by acoustic concentration) up to 48 hours after chemotherapy and subsequently a decrease at 72 hours. The changes observed in the QUS spectral parameters corresponded well with the trend of changes seen in the nuclear and cellular sizes and ratio, and number of nuclear fragments in the cell samples after treatment.

To measure the sensitivity of QUS parameters to detect apoptosis at this frequency, mixtures of apoptotic and untreated cells were made and imaged. Figures 4D-F demonstrate mean values obtained for the MBF, spectral slope and the 0-MHz intercept parameters acquired for mixtures of apoptotic and viable AML-5 cells with proportions ranging from 0 to 100% apoptotic cells. The maximum ultrasound backscatter power as represented by the MBF parameter occurs when 40–60% of the AML-5 cells were apoptotic. The MBF parameter increased by 10.9 ± 2.4 dBr on average between 0% and 40% mixtures of apoptotic cells. A similar pattern was observed with the spectral slope and 0-MHz intercept parameters. The spectral slope parameter increased from the baseline of 2.4 ± 0.3 dBr/MHz (0% mixture) to 3.7 ± 0.4 dBr/MHz at 40% apoptotic cell mixture. Similarly, the 0-MHz intercept demonstrated an average increase of 7.0 ± 1.9 dBr from 0% to 40% mixtures.

### *In vivo* experiments

**Figure 5:**
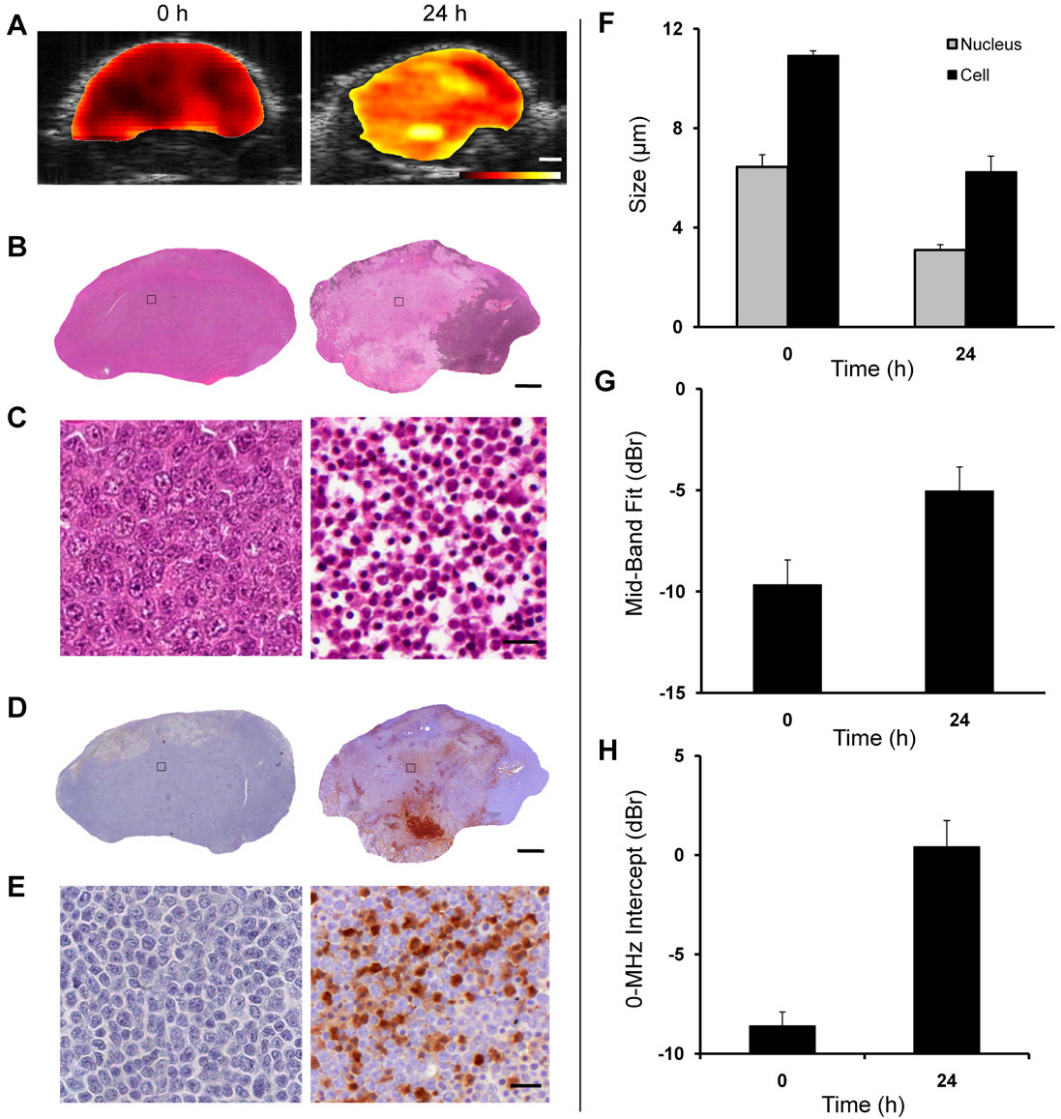
*In vivo* study on AML-5 tumour xenografts **A–E**: Representative data acquired from control and treated tumours. **A.** ultrasound B-mode images with ROI parametric overlays of the MBF parameter. The scale bar represents ~1 mm, and the color bar represents a scale encompassing ~40 dBr. **B, C.** light microscopy images of H&E stained slides at low and high magnifications. **D**, **E.** light microscopy images of TUNEL stained slides at low and high magnifications. The scale bar represents ~1 mm and ~20 μm in the low and high magnification images, respectively. **F.** Average values measured for the sizes of the cells and their nuclei before and after the treatment. **G, H.** Average values of the MBF and 0-MHz intercept parameters obtained before and after the treatment using ~7 MHz ultrasound. Error bars represent ± one standard error.

Chemotherapy produced appreciable and consistent changes in ultrasound backscatter and the spectral parameters *in vivo*. Increased backscatter power was observed in tumour xenografts 24 hours after chemotherapy exposure using low-frequency ultrasound. Results obtained via the *in vivo* study on AML-5 tumour xenografts are presented in Figure 5. Figure 5A demonstrates representative ultrasound B-mode images with ROI parametric overlays of the MBF parameter obtained before and after the treatment. The MBF parametric images demonstrate a considerable increase in ultrasound backscatter power within the tumour region in response to treatment. Figures 5B–5E present light microscopy images of H&E and TUNEL stained slides at low and high magnifications, respectively. Similar morphological and structural changes in cells and their nuclei as observed *in vitro* were also detected in the histology slides of the tumour xenografts. Specifically, nuclear condensation and fragmentation was readily detectable in the H&E stained slides after treatment. At higher magnification, extensive changes in morphology were observable in the treated area, including blebbing in the cellular membrane, nuclear coalescence and fragmentation, and structural disorganization. These alterations were a strong indication of dominant apoptotic cell activity that occurred in response to treatment, as also confirmed by the TUNEL staining. The TUNEL stained slides demonstrated areas of apoptotic cell death in treated tumours. This was clearly visualized in low and high magnification microscopic analysis. Average values measured for the size of the cells and their nuclei from the histology slides before and after the treatment have been presented in Figure 5F. Remarkable decreases are observed in size of cells and nuclei after the treatment, consistent with the observations *in vitro*. Figure 5G depicts mean values of the MBF parameter obtained before and after the treatment. The MBF parameter increased from −9.6 ± 0.2 dBr prior to treatment to −5.0 ± 0.2 dBr after treatment, a change that was found to be statistically significant (*t*-test, unpaired, two-sided, *p* < 0.05), compared to pre-treatment. Similarly, the 0-MHz intercept parameter demonstrated a statistically significant increase of 9.0 ± 1.5 dBr, on average, after treatment (Figure 5H). However, the spectral slope parameter did not show any statistically significant increases after treatment. The alterations in the size of cells and nuclei observed through the histological analysis corresponded well with the changes measured in the QUS parameters, specifically the MBF and the 0-MHz intercept.

## DISCUSSION

This study demonstrated the applicability of QUS spectral analysis techniques at conventional low frequencies to detect cell death *in vitro* and *in vivo* within hours after the start of treatment. Quantitative ultrasound spectral parameters derived from ultrasound RF data were demonstrated to be sensitive to changes in tissue micro-structure that are associated with cell death. The results were confirmatory across *in vitro* and *in vivo* studies where AML-5 cell samples and tumour xenografts underwent chemotherapy. As such, this study can be considered as an important extension to previous works that suggested a very good potential of QUS spectral analysis techniques at high-frequency ranges for evaluating cell death [11, 13, 14].

Particularly, time- and concentration-dependent *in vitro* experiments revealed changes in spectral parameters at conventional frequencies that were linked to cell death as indicated in standard histological analysis. Most chemotherapeutic agents such as cisplatinum mainly induce apoptotic cell death in tumour cells [28]. Apoptosis causes significant structural and morphological changes in cells and nuclei, which begins with nuclear coalescence, condensation, and fragmentation and eventually leads to nucleus degradation and loss [29, 30]. In our experiments, these changes were apparent in histological images of *in vitro* cell samples at different times after treatment. Nuclear condensation and fragmentation were observed up to 48 hours after treatment, whereas at 72 hours after treatment about half of the cells lost their nuclear structure. Also, average nucleus:cell size ratio decreased from 0.7 prior to treatment to 0.4 at 72 hours after exposure to cisplatinum. According to the results obtained, the MBF, spectral slope and the 0-MHz intercept parameters demonstrated changes that were consistent with these structural and morphological alterations in cells and their nuclei. In particular, these parameters demonstrated increases up to 48 hours after treatment and consequently start to decrease. Previous studies that applied QUS techniques at high-frequencies to detect cell death support the observations of this study with similar trends being identified albeit in more limited experiments [10, 11, 15]. Specifically, those studies demonstrated that early stages of apoptosis generally lead to large increases in ultrasound backscattered signal intensity acquired from *in vitro* cell aggregates [10, 13, 31] and *in vivo* tumour xenografts [14, 15]. However, it has been shown that the ultrasound backscattered signal intensity decreases in advanced stages of apoptosis [11, 32]. In particular, the early studies using high-frequency ultrasound showed that alterations in nucleus is the major cause for changes in ultrasound backscatter [11]. Early-stage cell-death (nucleus condensation and fragmentation) results in increases in ultrasound backscattered signal intensity. On the other hand, late-stage cell-death (chromatin dissolution/nucleus degeneration) has two contradictory effects on ultrasound backscatter. It initially increases the randomness in fairly regular distributions of scatterers, resulting in a larger backscattered signal. However, when a large fraction of the nuclei scatterers become disintegrated due to DNA cleavage (advanced necrosis), the amplitude of backscattered signal is reduced [11, 32, 33]. The results obtained from both time and concentration-based *in vitro* experiments in this study confirmed that a consistent trend is observed using QUS spectral parameters at conventional frequencies. The experiments conducted by mixing the two types of cells suggest an increase in backscatter from two effects. For 100% apoptotic cells an increase occurred purely as a result of the change in the nature of scatterers. For mixtures of viable and these same apoptotic cells where the backscatter was higher than that for the 100% sample it is possible that mixed scatterer types and their randomization contributes to the further increased backscatter detected. The effects of cell packing have been dealt elsewhere [10, 11] and a discussion on the nature of cellular causes of backscatter associated with cell death can be found in previous studies [15].

The changes observed in QUS spectral parameters with cell death progress *in vitro* were also similar to those obtained in the *in vivo* experiments. Particularly, the MBF and 0-MHz intercept parameters demonstrated a significant increase after chemotherapy in AML-5 tumour xenografts. Similar results were previously reported using high-frequency ultrasound *in vivo* [11, 13–15]. The spectral slope parameter did not show any statistically significant increases in AML-5 tumours *in vivo* after the treatment. The spectral slope parameter has been demonstrated to be linked to the size of acoustic scatterers in tissue micro-structures [16, 34]. Previous studies on cancer therapy response monitoring using high-frequency QUS techniques have shown that a time-dependent increase in spectral slope after treatment is correlated with changes in average nuclear size as results of apoptotic cell death [15]. In another study, however, Vlad *et al*. reported no considerable changes in the spectral slope parameter at high frequencies after radiotherapy, that induced both apoptotic cell death and mitotic arrest within AML-5 cells [13]. They explained this observation based on two opposite changes observed in size of potential acoustic scatterers due to nuclear condensation and fragmentations in apoptosis, and cell enlargement in mitotic arrest. In the context of conventional-frequency ultrasound applied in this study, bulk changes in tissue are believed to be related to ensembles of cells and nuclei smaller than the wavelength of the ultrasound being used. These ensembles influence acoustic properties and thus ultrasound backscatter characteristics. As such effects of different alterations in potential acoustic scatterers (cellular and nuclear condensation and fragmentation versus cell aggregation and development of patches of response) on the spectral slope parameter can be more likely superimposed, resulting in non-significant overall changes.

The results in this study demonstrated that development of cell death in response to anti-cancer therapies (in particular apoptosis in response to chemotherapy) could be detected by QUS techniques at conventional frequencies, as they result in detectable alterations in ultrasound backscatter power at these frequencies. These results are in correspondence with previous investigations performed *in vitro* and *in vivo* using high-frequency ultrasound in order to evaluate tumour response to various types of cancer-targeting treatments [11, 13–15]. Although both conventional and high-frequency ultrasound demonstrate similar trends of change in the spectral parameters in response to treatment, the predominant type of scattering expected at these two frequencies is potentially different. At the conventional frequencies, Rayleigh scattering (backscatter intensity proportional to f 4, where f is frequency) [35] is expected to predominate, since the cellular components are considerably smaller than the ultrasound wavelengths. In contrast, with high-frequency ultrasound, the cellular components are approximately proportional to the ultrasound wavelength, with a more complex relationship between backscatter intensity and frequency. As stated before, the ultrasound backscatter at conventional-frequencies is mainly impacted by bulk changes in ensemble of cells and nuclei, forming a speckle pattern of change due to alterations in bio-acoustic properties of cancerous cells after treatment. The interactions among the scatterers in cell ensembles have been shown previously to have a great impact on ultrasound backscatter properties at high frequency [32]. This can be an important factor at conventional frequencies as well.

The present study complements other imaging-based approach such as those based on magnetic resonance imaging or positron emission tomography being investigated for cancer therapy response monitoring [2, 5, 8]. Unlike these methods, the QUS techniques applied in this study rely on intrinsic contrast alterations arising from changes in bio-acoustic characteristics as cancer cells respond to treatment, hence do not require any injection of exogenous contrast agents. Ultrasound imaging has also the advantage of portability, low cost, rapid imaging speed, and high spatial resolution.

This study provides promise that QUS spectral analysis techniques can advance the monitoring of cancer treatment in the near future. QUS techniques at conventional frequencies applied here *in vitro* and *in vivo* can potentially be adopted for clinical use [36], permitting an early assessment of treatment response to cancer-targeting therapies on an individual patient basis. The research findings here form a basis for such applications of QUS spectral methods for therapy response monitoring.

## MATERIALS AND METHODS

### *In-vitro* experiments

#### Cell preparation and treatment

AML-5 cells (Ontario Cancer Institute, Toronto, ON, Canada) were cultured at a density of 3 × 105 cells/ml in in *α*-minimum essential medium (GIBCO 11900, Rockville, MD, USA) supplemented with 5% fetal bovine serum (Cansera International, Etobicoke, ON, Canada) and 1% penicillin/streptomycin (Invitrogen Canada Inc., Burlington, ON, Canada). The cells were maintained in 150 ml suspensions in tissue culture flasks (Sarstedt, Nümbrecht, Germany) in a 37°C thermo-incubator maintained at 5% CO. The cells were treated with 1.5 ml of cisplatinum, which is a chemotherapeutic agent that forms platinum-DNA adducts [19]. This disrupts DNA replication and transcription thus leading to apoptosis. The flasks were treated in sets of five, with a parallel set of untreated flasks (control), forming six sets of 0, 6, 12, 24, 48, and 72-hour time points. Treated AML-5 cells from 48-hour time-point set (100% apoptotic in this line) were mixed with untreated AML-5 cells to obtain proportions of apoptotic to healthy cells at 0%, 10%, 20%, 40%, 60%, and 100%.

At each time after treatment, two sets of six (treated and untreated) flasks were processed to form centrifuged samples for ultrasound imaging. Each flask was divided into 500 ml centrifuge containers and spun at 500 g in a fixed angle centrifuge at 4°C for 10 minutes. The condensed pellet of cells was re-suspended with phosphate buffer saline (PBS) and the concentrated suspensions were spun again in 500 ml conical tubes with a swinging bucket centrifuge at 1000 g. After a second re-suspension, the cells were spun once more at 2000 g in a three well (8 mm diameter) stainless steel container. The AML-5 samples mimic the close packing of cells as seen in solid malignancies. All samples (*n = 5* per group) were approximately the same size with a height of 5 mm each. Consistency of packing between the viable and apoptotic cells has been demonstrated previously [10].

#### Ultrasound data acquisition and analysis

Conventional-frequency ultrasound data were acquired with a Sonix RP (Ultrasonix, Vancouver, BC, Canada) system utilizing an L14–5/38 linear transducer with a center frequency of ~7 MHz, focused at 1 cm depth, and data sampled at 40 MHz. The AML-5 centrifuged samples were scanned at room temperature in stainless steel wells, at 6, 12, 24, 48, and 72 hours after exposure to cisplatinum. Ultrasound data were analyzed across multiple scan planes for each sample within a standardized region of interest (ROI), using an in-house software in MATLAB (MathWorks, Natick, MA, USA) and the results were averaged subsequently. The ROIs were determined manually on B-mode ultrasound images of the AML-5 sample in the well. The power spectrum was calculated using a Fourier transform of the raw RF data for each scan line through the ROI and subsequently averaged. The averaged power spectrum from each ROI was normalized to that of a reference signal from the base of a water-filled steel well, scanned with the same setting used for the cell sample scans, in order to remove system and transducer transfer functions. Linear regression analysis was performed on the normalized power spectrum data within a −6 dB window from the transducer center frequency, in order to provide a best-fit line. The MBF, 0-MHz intercept and spectral slope parameters were then calculated [13–17, 20–23] and reported for all samples.

#### Histological analysis

To investigate the morphology of cells, AML-5 cell samples were fixed with formalin, embedded in paraffin and stained with haematoxylin & eosin (H&E). Slides of AML-5 cells were prepared for the 0, 6, 12, 24, 48, and 72-hour time-point sets. Eighteen images from each slide were obtained at both 20× and 40× objective magnifications using a Leica DC100 microscope and a Leica DC100 camera connected to a 2 GHz PC equipped with Leica IM1000 software (Leica GmbH, Wetzlar, Germany). The sample on the slide was divided into 3 bands, *i.e*., top, middle, and bottom and 6 pictures of the AML-5 cells were taken from each band. The images were analyzed using ImageJ (National Institutes of Health, Bethesda, MD, USA), where a few thousand cells and nuclei were measured from untreated and treated slides. From the outset, 30 cells were measured from each image. Cell and nucleus size and the number of nuclear fragments (apoptotic cells) within each selected cell were measured. Cell and nucleus size was considered as the largest diameter of a cell or nucleus, respectively. Cells were selected if they had a nucleus and part or whole of the cell membrane was distinct from surrounding cells. Nuclei or nucleus fragments were measured (and counted) only if they were within a cell membrane. Terminal deoxynucleotidyl transferase dUTP nick end labeling (TUNEL) staining was also performed to confirm that the treated AML-5 cells were undergoing apoptosis.

### *In vivo* experiments

#### Ethics statement

This study has been conducted in accordance with the ethical standards, according to the Declaration of Helsinki and national and international guidelines, and has been approved by the authors' institutional review board.

#### Animal model and treatment

*In vivo* experiments were conducted on ten severe combined immunodeficiency disease (SCID) mice. One hind leg of each animal was subcutaneously injected with 106 in 50 μl of AML-5 cells. Tumours reached a size of 7–10 mm at 4–5 weeks after cell injection. The mice were then divided into two groups of untreated control and treated (*n = 5* per group). The treatment was administered using dexamethasone (0.246 mg/kg) [24] and cisplatinum (75 mg/m2) via intravenous tail vein injection.

#### Ultrasound data acquisition and analysis

All animals were imaged 24 hours after treatment, and subsequently sacrificed. Ultrasound data collection consisted of acquiring B-mode images in addition to raw RF data for quantitative spectral analysis of backscattered signal. After depilatory hair removal, animals were anaesthetized (Ketamine 100 mg/kg, Xylazine 5 mg/kg, Acepromazine 1 mg/kg; CDMV, St Hyacinthe, QC, Canada) and imaged using high viscosity ultrasound gel (ATL Inc., Reedsville, PA, USA) as coupling agent over the skin in tumour-bearing areas. The same ultrasound system and transducer was used as applied in the *in vitro* experiments. The ROIs (approximate size of 5 × 5 mm) were located at the tumour center which was consistently positioned at the transducer focal depth (1.5 cm). The same spectral analysis method was used as described in the *in vitro* experiments with an glass-bead-embedded agar-gel phantom model [25, 26] used in place of stainless steel as a reference, and scanned with the same setting as used for the tumour scans. Ten to fifteen ROIs were analyzed for each tumour sample and the results were averaged subsequently. In addition to the averaged QUS parameters, parametric images of these ultrasound-based biomarkers were also generated for a visual representation of the RF data analysis. Parametric images were generated utilizing a sliding window analysis within the ROI using a Hamming function. The size of the sliding window was selected to cover approximately 10 wavelengths in order to obtain robust parametric maps independent of the window length [27].

#### Histological analysis

After imaging and sacrificing the animals, the tumours were excised and perfused with paraformaldehyde in order to facilitate immunohistological analyses. For each animal, four representative tumour sections were stained using H&E staining and TUNEL immunohistochemistry as above for *in vitro* samples. Light microscopy was carried out at 20× and 40× objective magnifications, and digital images were analyzed using ImageJ, which was used to visualize the total tumour areas and apoptotic cell death areas from TUNEL stained images. Moreover, apoptotic cells were identified manually at higher magnification by recognizing typical apoptotic bodies. Cell and nucleus sizes were also measured at high magnification in H&E stained images within multiple representative tumour regions.
